# Topological Data Analysis Highlights Novel Geographical Signatures of the Human Gut Microbiome

**DOI:** 10.3389/frai.2021.680564

**Published:** 2021-08-18

**Authors:** Eva Lymberopoulos, Giorgia Isabella Gentili, Muhannad Alomari, Nikhil Sharma

**Affiliations:** ^1^ Department of Clinical and Movement Neurosciences, Institute of Neurology, University College London, London, United Kingdom; ^2^ CDT AI-Enabled Healthcare Systems, Institute of Health Informatics, University College London, London, United Kingdom; ^3^ R^2^ Data Labs, Rolls-Royce Ltd, Derby, United Kingdom; ^4^ National Hospital for Neurology and Neurosurgery, University College London Hospitals NHS Foundation Trust, London, United Kingdom

**Keywords:** gut microbiome, human microbiome, population health, global variation, topological data analysis (TDA)

## Abstract

**Background:** There is growing interest in the connection between the gut microbiome and human health and disease. Conventional approaches to analyse microbiome data typically entail dimensionality reduction and assume linearity of the observed relationships, however, the microbiome is a highly complex ecosystem marked by non-linear relationships. In this study, we use topological data analysis (TDA) to explore differences and similarities between the gut microbiome across several countries.

**Methods:** We used curated adult microbiome data at the genus level from the GMrepo database. The dataset contains OTU and demographical data of over 4,400 samples from 19 studies, spanning 12 countries. We analysed the data with *tmap*, an integrative framework for TDA specifically designed for stratification and enrichment analysis of population-based gut microbiome datasets.

**Results:** We find associations between specific microbial genera and groups of countries. Specifically, both the USA and UK were significantly co-enriched with the proinflammatory genera *Lachnoclostridium* and *Ruminiclostridium*, while France and New Zealand were co-enriched with other, butyrate-producing, taxa of the order Clostridiales.

**Conclusion:** The TDA approach demonstrates the overlap and distinctions of microbiome composition between and within countries. This yields unique insights into complex associations in the dataset, a finding not possible with conventional approaches. It highlights the potential utility of TDA as a complementary tool in microbiome research, particularly for large population-scale datasets, and suggests further analysis on the effects of diet and other regionally varying factors.

## Introduction

In recent years, there has been a rapidly growing interest in the connection between the gut microbiome and disease. This area spans detailed exploration of the gut microbiome in small specific clinical disease phenotypes to larger population-level studies. In parallel, there have been advances in the analytics approaches the microbiome field has adopted to test the different hypotheses. Conventional approaches employ dimensionality reduction and typically assume linearity. Here we use topological data analysis (TDA) exploring the difference and similarities between the gut microbiome across several countries. We highlight unique insights that are made possible with the use of TDA.

### The Human Gut Microbiome

The gut microbiome is a diverse community of an estimated 100 billion to trillion microorganisms - bacteria, viruses, and fungi - inhabiting the intestine and gut (Sender et al., 2016). So far, 1,952 species have been classified - however, the majority of the microbiome remains unreferenced (Almeida et al., 2019). Unsurprisingly, the relationships between the different microbiome species are highly complex, dynamic, and nonlinear (Shoaie et al., 2013). Depletion of one species below a specific threshold can lead to the so-called blooming of others. Some species also exist only in either very low or very high abundance with specific tipping points (Lahti et al., 2014). Species can even change their phenotype based on the concentration in the gut, environmental, or genetic context; in other words, harmless bacteria can become pathogenic under specific circumstances (Casadevall, 2017). These species have so-called high pathogenic potential, are also known as pathobionts, and are usually kept under control by a healthy microbial community (Kamada et al., 2013). If this ecosystem is disrupted, pathobionts and external pathogens can bloom, affecting host health. It is important to note that a healthy composition of the microbiome is highly individual but based around a proposed universal “core microbiome” (Rinninella et al., 2019).

The microbiome coevolved with humans in a commensal, perhaps even symbiotic, way (Bäckhed et al., 2005; Shapira, 2016). The microbiome appears to play a central role in host immunity, metabolism, behaviour, and cognition through yet unclear pathways. Specifically, it is thought that a disturbed microbiome, also called gut dysbiosis, can set off inflammatory cascades. Disease, lifestyle changes, or environmental influences can disturb the delicate balance of the microbiome, leading to loss of seemingly beneficial microbes and a simultaneous blooming of bacterial taxa detrimental to the host (Petersen and Round, 2014). This can lead to the breakdown of the epithelial cells lining the gut, increasing gut permeability which can cause pro-inflammatory bacterial metabolites or products to leak out, triggering further inflammatory cascades in the host (Rooks and Garrett, 2016; Thevaranjan et al., 2017). Changes in the gut microbiome have increasingly been linked to a range of diseases, such as colitis, diabetes, neurodegenerative diseases, and autism (for a review *see* Ghaisas et al., 2016)). Additionally, the gut microbiome influences the efficacy and bioavailability of oral medication (*see* e.g. Enright et al., 2016; Wilson and Nicholson, 2017; Clarke et al., 2019). For instance, the interaction between drugs and microbiome appears important in Parkinson’s disease (Rekdal et al., 2019), arthritis (Scher et al., 2020), schizophrenia (Seeman, 2021), and bipolar disorder (Flowers et al., 2020). Analysing the gut microbiome and illuminating the subtle relationships driving it has significant translational value for population health, particularly as it is an easily accessible and scalable potential therapeutic target.

### Variation in the Gut Microbiome

Several factors affect the gut microbiome, which can be broadly distinguished into lifestyle, medical, and environmental factors. Perhaps the most prominent lifestyle factor is diet. A high-fat diet can induce dysbiosis in the gut microbiome (Vaughn et al., 2017), while a diet high in resistant starches and complex carbohydrates (such as the Mediterranean diet) increases beneficial species (Garcia-Mantrana et al., 2018). This includes Firmicutes that produce short-chain fatty acids (SCFA), which have anti-inflammatory properties and maintain the integrity of the epithelial layer in the intestine (Morrison and Preston, 2016; Levy et al., 2017). Similarly, moderate alcohol consumption seems to increase anti-inflammatory species (Quesada-Molina et al., 2019), and exercise is also associated with a beneficial effect (Monda et al., 2017). Travel has also been shown to negatively alter the microbiome by decreasing diversity (Riddle and Connor, 2016; Langelier et al., 2019). Crucially, hygiene is a non-negligible factor - while inadequate sanitation can increase the likelihood of bacterial infection, excessive hygiene as practised in some countries - even as a response to the COVID-19 pandemic - may lead to a reduction in the microbiome diversity (Schmidt et al., 2011; Burchill et al., 2021).

The use of oral medications, particularly antibiotics, is another important influence on the gut microbiome. It takes some microbial species up to 6 months to recover from a complete cycle of antibiotics (Dethlefsen et al., 2008). Non-antibiotic medication such as dopaminergic drugs (Hill-Burns et al., 2017), proton pump inhibitors, antipsychotic drugs, and opioids interact with the microbiome and can affect its composition (Le Bastard et al., 2018). As the microbiome is interlinked with metabolic pathways, chronic diseases such as diabetes are also associated with a disrupted gut microbiome, though the causal direction of this effect is unclear - the same holds true for obesity (Singer-Englar et al., 2019). Environmental factors are a crucial and sometimes overlooked part of the host-microbiome relationship. External pathogens such as viruses can induce changes to the gut microbiome, as can pesticides and other toxins (Li N. et al., 2019; Tu et al., 2020). Pollution has also been associated with changes to the microbiome, particularly air pollution (Vallès and Francino, 2018; Bailey et al., 2020). Crucially, there is evidence that the soil and drinking water microbiomes interact with the gut microbiome (Blum et al., 2019).

### Geographical Variation of the Gut Microbiome

The factors influencing the microbiome vary regionally, leading to differences in the population microbiome across countries as has been observed in many past studies (e.g., Karlsson et al., 2014). One large review reports distinct geographical differences in the gut, oral, and skin microbiomes between non-industrialised and industrialised populations in addition to a conserved core microbiome (Gupta et al., 2017). More specifically, the review reported that while the non-industrialised gut microbiota include more species of the phyla Proteobacteria, Spirochaetes, order Clostridiales, and genera *Prevotella* or *Ruminobacter*, the industrialised communities were more enriched with the Firmicutes phylum, and *Bacteroides* and *Bifidobacterium* genera.

Diet is one of the most intuitive drivers of these differences; while some countries consume large amounts of meat, others have a diet heavier in carbohydrates, or in fibre (Ritchie and Roser, 2019), which has been connected to observed differences in microbiome composition between countries (Riaz Rajoka et al., 2017). For example, one study compared gut microbiome signatures between children in urban Italy and rural Burkina Faso and found unique microbial genera in the African children that might be linked to differences in diet: the genera *Prevotella*, *Xylanibacter*, *Butyvibrio*, and *Treponema* are involved in cellulose and xylan hydrolysis which are fitting for the polysaccharide-rich diet of the African children which includes many whole grains, producing the beneficial SCFAs (De Filippo et al., 2010). These results are echoed in a later study comparing Egyptian and US-American teenagers, which found differences in the metabolic profiles consistent with the dominant diet of the respective region (Shankar et al., 2017).

Similarly, prescription patterns and access to antibiotics vary from country to country: while low- and low-middle-income countries count around 12 daily antibiotic doses per 1,000 citizens, high-income countries count around 25 per 1,000 (Klein et al., 2018). Pesticide use is another factor that varies starkly between countries, due to environmental regulations being less or more restrictive, as well as the importance of farming or industry for a country’s economy and society (Handford et al., 2015). Accordingly, soil and water microbiome signatures vary between countries and regions, as demonstrated by the Earth Microbiome Project (Thompson et al., 2017). This is partly naturally caused, and partly due to external factors such as pesticide and fertiliser use (Gourmelon et al., 2016; Lupatini et al., 2017). In addition to these environmental factors, host genetics and the innate and adaptive immune systems can account for some of the human microbiome variation between populations, although the exact contributions of environmental and genetic factors, respectively, are unclear (Gupta et al., 2017).

Together, these factors could point to differences in the population microbiome which are important to health and disease. As some of the differences between populations described above include increased anti-inflammatory microbial products, this can affect inflammatory and disease processes in these regions. One example of this has been research into obesity: while obesity varies between countries and has been connected to the industrialisation level of a population, it has also been associated with a differential microbiome profile (Dugas et al., 2016). Mouse studies have even suggested causality: transplanting the gut microbiome of genetically modified obese mice into germ free mice led to weight gain (Turnbaugh et al., 2006). However, human data on whether shifts in the microbiome associated with geographical variations relate to geographical differences in obesity are rare. One recent study found that the gut microbiome of obese subjects in industrialised countries is more similar to that of other industrialised countries, even if these were geographically far apart, than to that of non-industrialised communities (Angelakis et al., 2019). Similar geographical insights could be relevant for non-communicable diseases that have been associated with deviations in the microbiome and that have differential prevalence in some countries over others, as has been observed for many gastrointestinal, neurodegenerative, psychiatric, or inflammatory diseases (GBD 2017 Disease and Injury Incidence and Prevalence Collaborators et al., 2018). Knowledge about what drives these differences could in turn inform improvements to existing medications or inspire novel treatment options through the gut microbiome.

### Limitations of Standard Analysis

Traditional approaches to microbiome analysis comparing groups, even ones employing complex machine learning models, have many shared limitations, preventing reliability. Firstly, they rely on reduction in dimensionality to simplify the modelling of the ecosystem, which leads to loss of key information around the complex interplay of the microbiome. The binary output of these studies, namely which taxa are deemed to be beneficial or detrimental, is an oversimplification of the original problem. Attempting to address the highly complex and non-linear ecosystem of the gut microbiome with a simplistic linear approach introduces a range of errors to the results, such as precluding the real effect and leading to frequent false positives if not adequately addressed. Additionally, many human microbiome studies, including the ones on geographical variation, have very small sample sizes, particularly those comparing patients to healthy controls. Many also poorly control for potential confounders. There have been efforts to curate larger datasets to tackle some of these issues, leading to sample sizes of up to 12,000 in the American Gut Project (McDonald et al., 2018). However, the issues cannot be countered with an increase in sample size on its own - in fact, it can be argued that adding more data while maintaining the oversimplified, linear modelling approaches will add further noise to the results and lead to multiple comparison errors.

### TDA and the Microbiome

Topological data analysis (TDA) can address many of these concerns. TDA is an analysis method coined by Gunnar Carlsson (2009) and was developed to analyse high-dimensional datasets. It uses principles from topology and differential geometry, specifically persistent homology. By doing so, TDA can represent the underlying geometric structure, or shape, of the data while accounting for its complexity. Additionally, TDA deals well with high-throughput biological data, such as the microarrays used to sequence the microbiome. It is therefore designed to detect subtle and non-linear relationships in the data and can deal with noisy or incomplete datasets. These factors support the use of TDA in microbiome research.

One previous study by another group has demonstrated the value of TDA for microbiome analysis by combining the well-known Mapper (Singh et al., 2007) with the Spatial Analysis of Functional Enrichment (SAFE) algorithm (Baryshnikova, 2016) to detect co-variance between metadata and microbiome taxa in the dataset (Liao et al., 2019). The authors report that *tmap* outperformed standard tools such as *envfit*, *adonis*, and ANOSIM in a synthetic dataset, specifically in detecting non-linear, as well as mixed non-linear and linear associations within the data. They applied *tmap* to two population-based microbiome datasets, the Flemish Gut Flora Project (Falony et al., 2016) and the American Gut Project which further illustrates the potential to detect non-linear relationships, specifically associations with host-metadata. They report co-enrichment between two of the so-called enterotypes (Arumugam et al., 2011) and countries, specifically the USA with the Bacteroidetes enterotype and the UK with the Ruminococcaceae enterotype. Further analyses revealed co-enrichment of diet and medication, as well as other lifestyle factors, which were thus associated with both the countries and the enterotypes. TDA appears to be a promising tool to investigate the microbiome through large population-based datasets, specifically as it highlights the increased signal detection in noisy data. While an important and powerful proof of concept, a key scientific limitation of this study was the comparison of only two countries, limiting conclusions on geographical variation of the microbiome that can be drawn from this. Additionally, the authors did not investigate specific underlying microbiome taxa but focused instead on enterotypes, potentially missing more subtle relationships.

### This Study

This present study aims to explore the relationship between a range of countries and specific microbiome signatures using TDA. To this end, we use a large repository of gut microbiome data spanning 12 countries with over 4,400 samples and apply the TDA pipeline *tmap* to investigate the co-enrichment of countries and specific microbiome taxa. To our knowledge, this is the first study using this analysis pipeline for this purpose on this data. We hypothesise that with this approach, we can find evidence for differences but also similarities in the gut microbiome signatures that have previously been overlooked by conventional microbiome approaches. This is important in developing our understanding of the microbiome not as a combination of singular taxa but as a rich, diverse, and interrelated ecosystem.

## Methods

### Dataset

Microbiome data is obtained from stool samples that are metagenomically sequenced, and then taxonomically classified. The data is thus stored as operational taxonomic units (OTUs).

For this study, we used data from GMrepo, a database of curated gut microbiome metagenomes (Wu et al., 2020). Using the provided RESTful API, we obtained all run IDs associated with the “healthy” and adult phenotype (Mesh-ID D006262) and filtered for only those samples that passed quality control. We then used the run IDs to download the full metagenomic sequence at the genus level. Countries with less than 20 samples were excluded. Metadata of interest that were collected for the whole sample are age, sex, and BMI. BMI was coded into underweight (BMI below 18.5), normal (18.5–24.9), overweight (25–29.9), and obese (over 30) according to the criteria adopted by the WHO, NIH, and NHS.

### Analysis Pipeline

Data analysis was conducted in Python 3.6, in a Jupyter notebook 6.0.2 environment. The scripts are available from thesharmalab.com GitHub repository.

A key aspect of TDA approaches is the production of the underlying shape and persistence of the structures. To explore this, we first produced a persistence diagram on the microbiome data as a point cloud with the giotto-tda package (Tauzin et al., 2021). This could then inform parameter tuning during subsequent steps. Then, TDA was conducted with the tmap analysis pipeline (Liao et al., 2019). The pipeline is an “integrative framework” based on TDA and is specifically designed for stratification and association analysis of population gut microbiome datasets. It utilises two established algorithms for TDA and stratification analysis, the Mapper and SAFE algorithms, respectively.

### TDA With Mapper

The input to the Mapper algorithm is a point cloud of data points, in this case, each data point represents one stool sample. First, pairwise distances are calculated with the Bray-Curtis distance and these are then transformed to a square-form distance matrix. This matrix is filtered from the original high-dimensional space into a low-dimensional space using multidimensional scaling (MDS), a non-linear method of dimensionality reduction which translates pairwise distances among data points into the low-dimensional space (Mead, 1992). This filter was used as in the origination of the Mapper algorithm (Singh et al., 2007), and the components were set to two, as recommended by the developers of the *tmap* pipeline, with the “pre-computed” metric. Next, the low-dimensional space is partitioned into bins using overlapping covers with each cover including a subset of data points that overlap in some way. Within each cover, data points are then clustered based on the distances from each other in the original, high-dimensional space. These clusters are represented as a node in the TDA network. The shape of the network is a combination of distances in the low- and high-dimensional spaces. In other words, each node in the network is a group of samples with overlapping microbiome profiles and each link between the nodes indicates a shared sample between nodes. The clusterer used was the Density-Based Spatial Clustering of Applications with Noise (DBSCAN) from scikit-learn, as is recommended in tmap documentation. To set an appropriate maximum distance between two data points (*eps*), we used the Mapper algorithm automated optimisation function (optimize_dbscan_eps) with a threshold of 95%, which specifies the percentage of samples for which to cover or cluster the surrounding neighbourhood, based on the distribution of nearest-neighbour distances. The minimum number of neighbours was set to 5.

**FIGURE 1:**
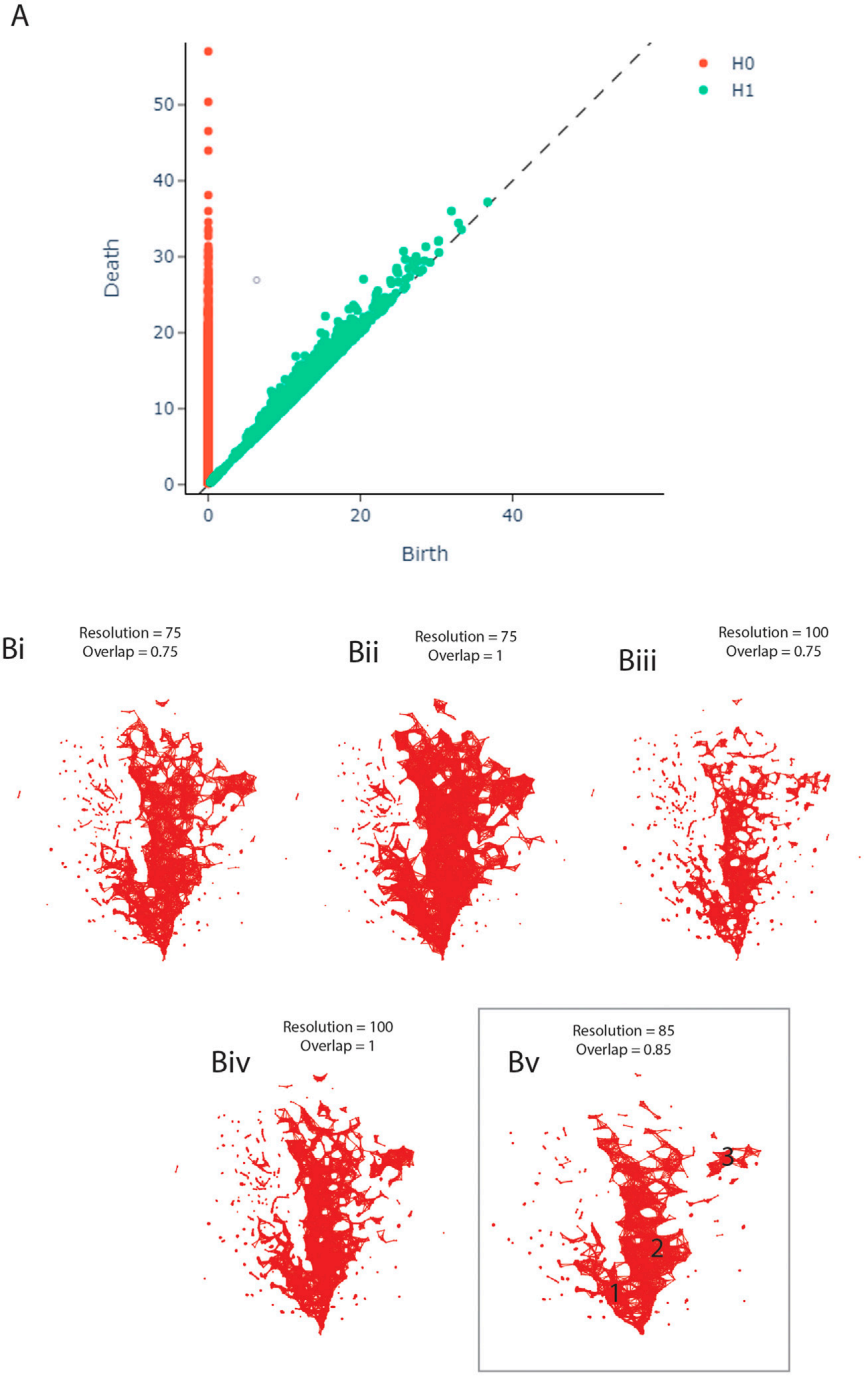
**(A)** Persistence Diagram. **(B)** Adjusting the resolution and overlap parameters of the TDA network to achieve optimal cover ratio. Bv shows the final network, including the three clusters.

To optimise the cover ratio, a measure of how many samples are retained during the clustering process, the resolution and overlap parameters were adjusted. Resolution is a measure of how many bins the data is being split into, while overlap decides how big the overlap between adjacent bins needs in order to be considered overlapping. Resolution determines how sparse versus coarse the network will be and thereby how many nodes the network will have. Overlap, on the other hand, determines how densely connected the network will be and thereby how many edges the network will have. Both parameters were adjusted by hand and are shown in Figure 1B.

### Enrichment With SAFE

The SAFE algorithm maps values of a variable onto the network, denoting enrichment of this variable. The algorithm uses the TDA network as input and then maps the values of a given variable onto the network as node attributes. For example, if the variable is age, then the SAFE algorithm maps the average age of each node (i.e., group of samples). This is called network enrichment.

Subsequently, each node is examined in subnetwork analyses while permutating a given number of times over the entire network which determines how significant the observed enrichment is. For this study, the number of network permutations during this step was set to 5,000 to maximise sensitivity. A subnetwork is identified as a local neighbourhood around each node, where constituent nodes are selected according to the maximum distance threshold. We kept this threshold at the 0.5th percentile of all pairwise node distances in the network. For each neighbourhood, the enrichment of observed values at the neighbour nodes is summed and then ranked to compare the observed with the permutated scores. The score is then log-transformed and normalised to yield a so-called SAFE score for each node of the network. To reiterate, the SAFE scores quantify the enrichment level of a variable in the nodes around a given node. These local scores can be filtered and summed to yield the SAFE enriched score, which represents the network-level association of a target variable. It can be used as analogous to an effect size, allowing comparison between variables in the form of ranking, as well as investigation of their co-enrichment of variables.

Stratification, meaning subgrouping of a population, can be conducted by analysing the enrichment of a host metadata variable across the network. For this, metadata and taxa were entered as covariates for the network enrichment analysis described above. Continuous data, such as age and the microbial taxa, yield stratification heat maps. These show the distribution of absolute values across the network (with the mean of each group of samples represented as one node value), as well as the distribution of enrichment across the network as represented with the SAFE score for each node. Note that dark blue corresponds to the number 0 in both cases. All countries, as well as the metadata variables sex and BMI, were dummy coded. The different levels, or groups, of each variable can be plotted against each other by comparing the SAFE scores of each level at a given node. This means that for each node, the visualisation shows which group was more enriched. If none of the groups show enrichment at a given node, it is grey. Additionally, the most enriched taxa can be found by identifying the most enriched taxon of each node and colouring that node accordingly. It is important to note that to assess significance of an enrichment, the SAFE algorithm depends on both sample size and distribution across the TDA network, affecting the SAFE score, number of significantly enriched nodes, and the SAFE enriched score. This means that for a metadata variable with few samples that are highly distributed across the network, the probability of permutation is very small, making assessment of the enrichment difficult. This affects interpretation of the results, especially for countries with low sample size. If these countries have low SAFE scores, this does not signify the absence of an effect; instead, it demonstrates an inability to detect the presence of an effect. This should be kept in mind when interpreting the results of the SAFE algorithm.

Finally, co-enrichment between variables can be determined, which describes relationships between host metadata and microbiome variations (Liao et al., 2019). While two variables can be considered co-enriched if they enrich in the same area of the network – suggesting that they account for the shape of the network in this area –, it is also possible to quantify this association. For this, we calculated the pairwise co-enrichment for all taxa and metadata, yielding the significance level of each pair. We then applied a threshold to the significance at the 0.5th percentile and binarized the data accordingly. This strict threshold was used to account for the large number of pair-wise tests and reduce the type I error rate. The binarization allowed us to easily find significant co-enrichment between variables. Specifically, we used this quantitative indicator to supplement visual indications of co-enrichment, such as enrichment in the same areas of the network, between the variables most highly enriched across the network.

## Results

### Dataset

Based on our criteria, the final dataset includes 4,437 stool samples, 1,341 taxonomic units, as well as relevant study and host metadata. The data spans 12 countries from 19 studies, including both Amplicon and metagenomic data. Mean and standard deviations for age, as well as the distribution of sex and BMI for each country, are shown in Table 1.

**TABLE 1:** Demographic Data.

	Brazil	Canada	China	Denmark	France	Germany	Italy	New Zealand	Spain	Tanzania	UK	USA	Total
**Age**													
Mean	30.1	25.9	43.3	55.4	62.0	38.1	39.3	36.9	40.9	36.1	51.3	41.7	**40.0**
SD	5.0	5.1	12.4	8.1	10.5	8.3	13.6	12.6	14.5	13.3	13.2	16.7	**16.9**
**Sex**													
Female	18	659	81	73	249	0	26	82	31	8	122	847	**2196**
Male	2	610	90	34	216	70	14	49	16	14	149	965	**2229**
Missing	0	0	0	0	0	0	0	0	0	0	2	10	**12**
**BMI**													
Underweight	0	0	8	0	4	0	1	0	0	0	8	38	**59**
Normal	15	973	33	1	228	29	26	101	2	0	180	1059	**2647**
Overweight	4	296	32	0	182	29	2	30	0	0	69	495	**1139**
Obese	1	0	0	0	38	12	0	0	0	0	16	95	**162**
Missing	0	0	98	106	13	0	11	0	45	22	0	135	**430**
**Total**	**20**	**1269**	**171**	**107**	**465**	**70**	**40**	**131**	**47**	**22**	**273**	**1822**	**4437**

### Persistence Diagram

The persistence diagram (Figure 1A) shows four highly persistent structures in dimension 0 - representing clusters - and no high persistence in dimension 1 - representing loops. We thus expect to see two to four clusters and a relatively noisy network in the next step of our analysis.

### Parameter Adjustment

During the construction of the TDA graph, the resolution and overlap parameters were adjusted by hand to obtain the optimal cover ratio that is representative of the persistence diagram, meaning two to four clusters and no loops. The panels in Figure 1B show the result of this adjustment. The final network was constructed with the resolution set to 85 and overlap to 0.85 (Figure 1Bv).

### TDA Network

The TDA network produced by tmap contains 1,435 nodes and 8,870 edges, based on 2,910 samples. 1,527 (65.58%) samples had to be dropped during the construction of the network, likely due to missing data as the individual studies did not measure the same taxa, leading to many OTUs being marked as 0 in each sample. As can be seen in Figure 1Bv, the network has two central clusters, a smaller one on the left (1) and a larger one in the middle (2). There is also a small third cluster on the right (3). This pattern is broadly consistent with the persistence diagram (Figure 1A).

### Geographical Enrichment

**FIGURE 2:**
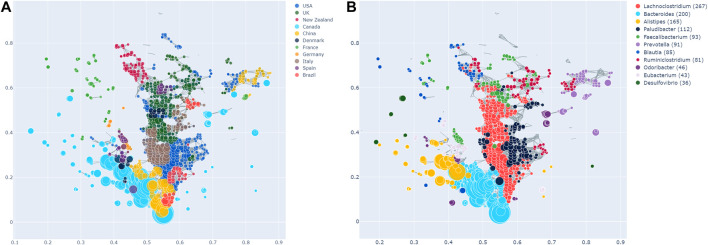
**(A)** Network stratification of the countries in the dataset, showing the enriched nodes. Note that Tanzania is not included here, as it did not have any significantly enriched nodes. However, this does not necessarily imply the absence of an effect. **(B)** Network stratification of the taxa with the most enriched nodes.

Enrichment of the countries across the TDA network is shown in Figure 2A, in which each node is coloured according to which country has the most enrichment at that local node. Additionally, larger nodes correspond to a larger number of samples in that node.

Most countries are either predominantly enriched in cluster 1 (e.g., Canada) or cluster 2 (e.g., the USA, the UK, Italy, New Zealand). Interestingly, the USA and the UK are also significantly co-enriched. China is enriched at the junction of cluster 1 and 2, as well as in cluster 3, in which they are together with the USA and Canada. Brazil is enriched both in cluster 2, as well as the junction of the two big clusters. Samples from France are enriched in the same area of the graph, namely the top left, which appears sparse and disconnected from the rest of the network. As can be seen in Figure 1B, this holds true for all the observed parameter adjustments. Finally, Tanzania is not significantly enriched in the network at all. As mentioned above, this finding needs to be interpreted cautiously due to the low sample size of Tanzania.

**FIGURE 3:**
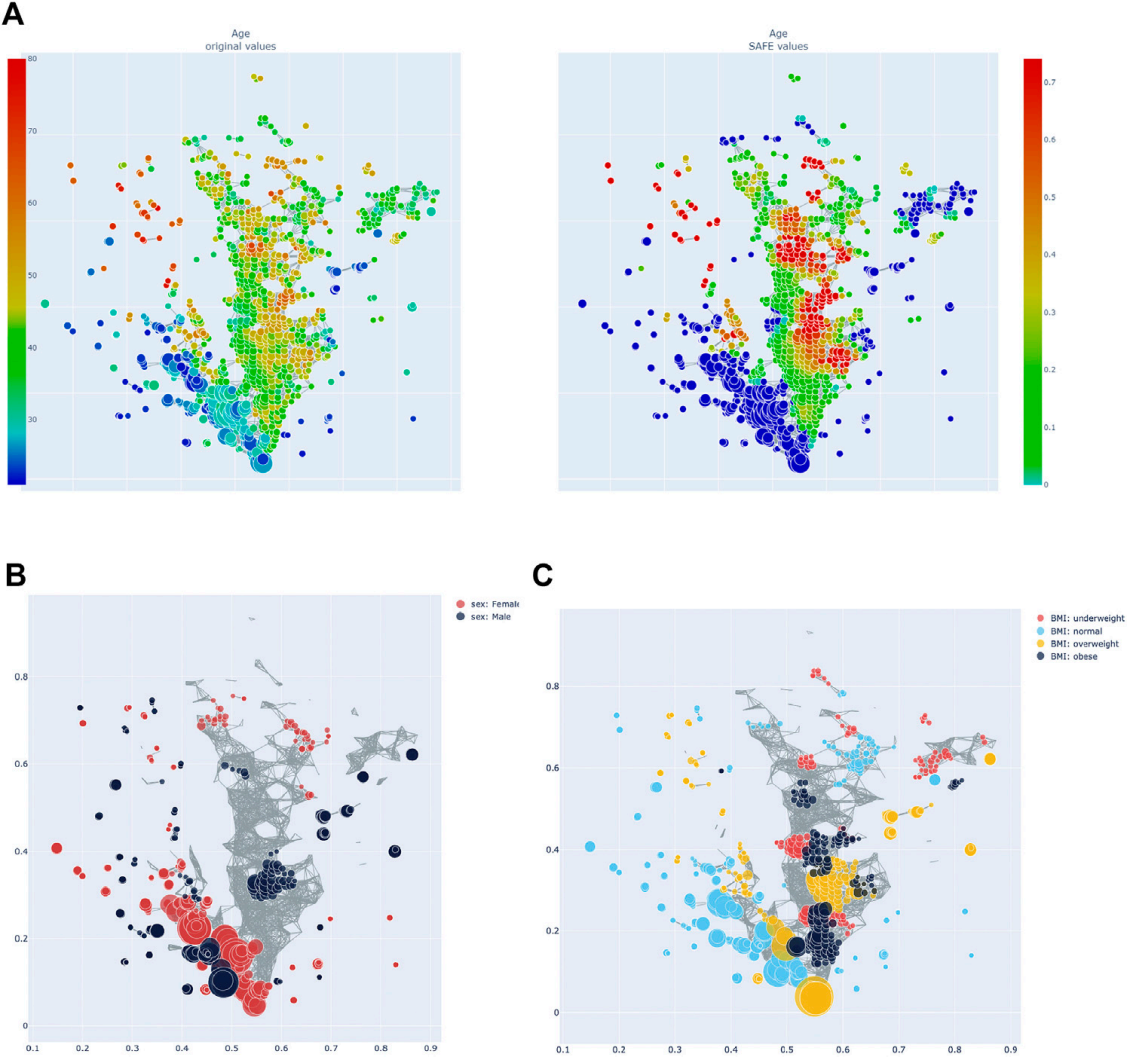
**(A)** Heatmap of age: distribution of absolute value across the network on the left, enrichment on the right. **(B)** Network stratification of sex across the network. **(C)** Network stratification of BMI across the network.

Figure 3A shows all host metadata ranked according to their SAFE enriched scores. The USA and Canada stand out with scores of over 400 each, making them the two most enriched host metadata. The next most enriched country is the UK with a SAFE score of 190, and China with a score of 84. Together with France, these countries also have the most samples (*see* Table 1).

### Other Metadata

**FIGURE 4:**
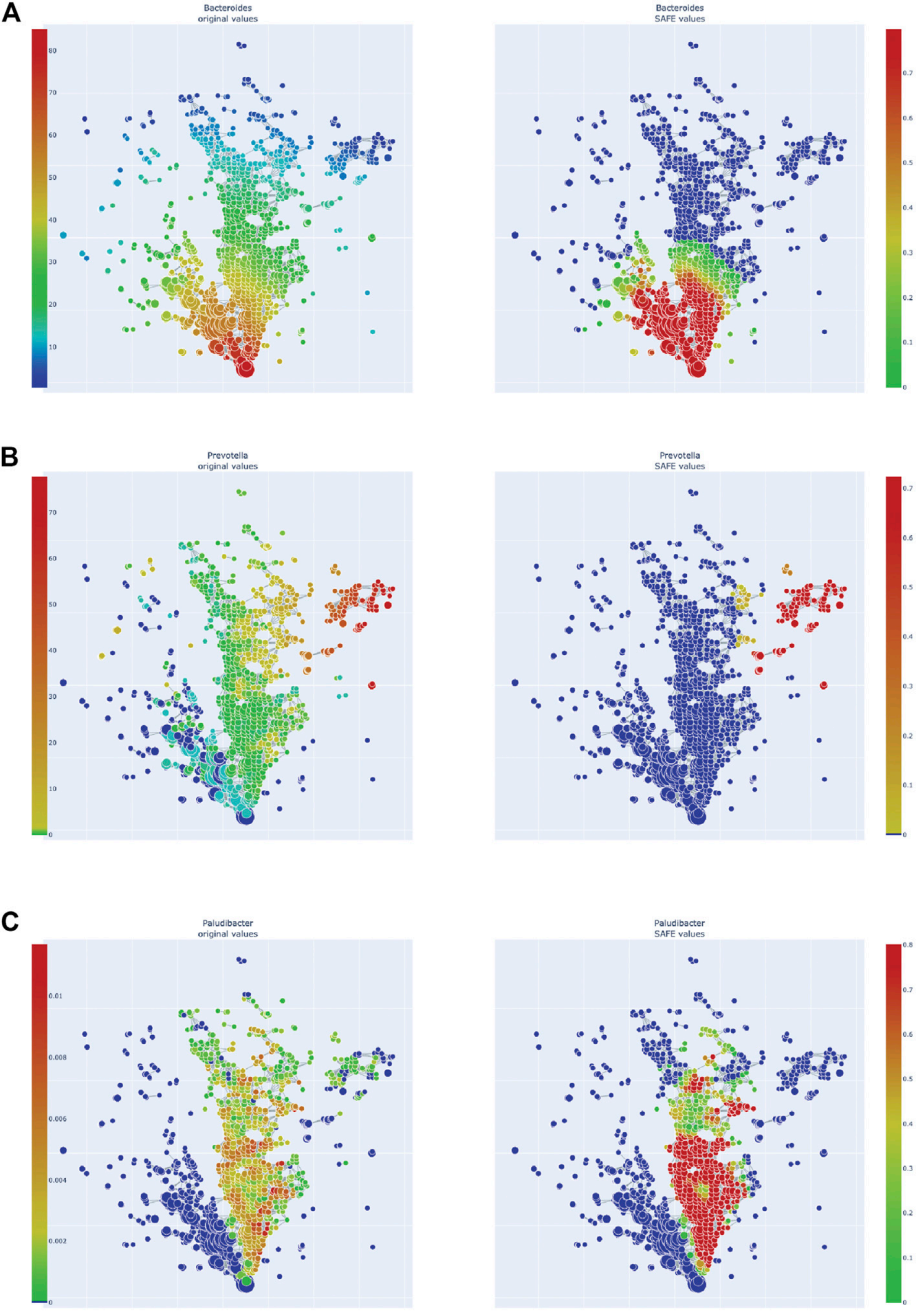
Enrichment heatmaps of three Bacteroidetes genera, absolute abundance across the network on the left, enrichment on the right. **(A)** *Bacteroides*. **(B)** *Prevotella*. **(C)** *Paludibacter*.

Age, sex, and BMI were also investigated. Host age has a SAFE enriched score of 205 and is enriched mostly in cluster 2 (*see* Figure 4A). Host age is significantly co-enriched with the UK and France, which both have higher than average age compared to the other countries (*see* Table 1). Host sex was relatively highly enriched (SAFE enriched scores Male: 223, Female: 210), and the enrichment network shows that female sex is mostly enriched in cluster 1, while the enrichment of male sex is more distributed across the network (Figure 4B). Interestingly, both female and male sex are significantly co-enriched with Canada. Finally, BMI seems to be a relevant host variable, as normal BMI is the third most enriched metadata with a SAFE enriched score of 302. Most of the enrichment of the normal BMI appears in cluster 1, while cluster 2 is more enriched with non-normal BMI phenotypes (Figure 4C). This is reflected in a significant co-enrichment of normal BMI with Canada. Further, normal BMI is significantly co-enriched with male sex.

### Taxa

**FIGURE 5:**
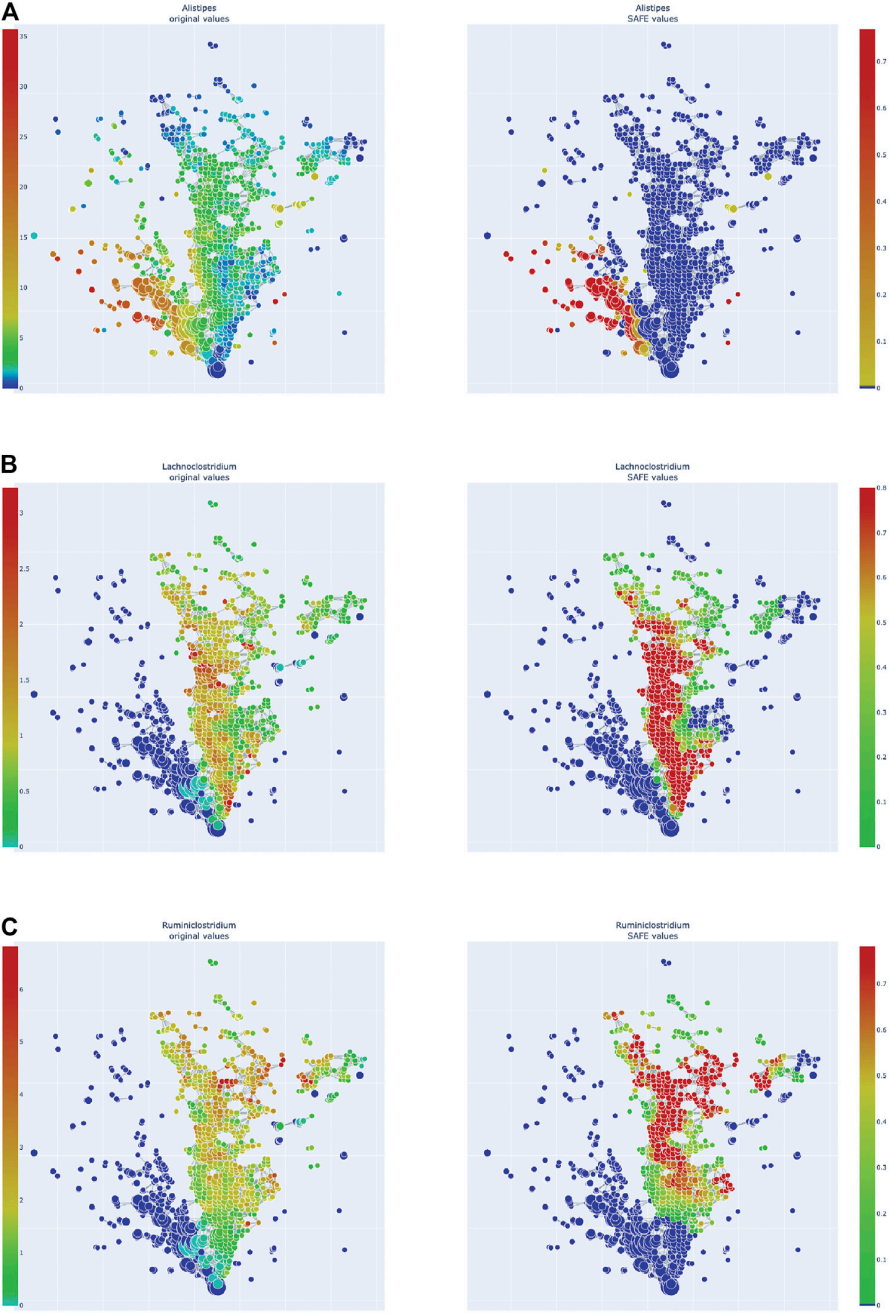
Enrichment heatmaps of one Bacteroidetes genus and two Firmicute genera, absolute abundance across the network on the left, enrichment on the right. **(A)** *Alistipes, of Bacteroidetes phylum*. **(B)** *Lachnoclostridium*. **(C)** *Ruminiclostridium*.

**FIGURE 6:**
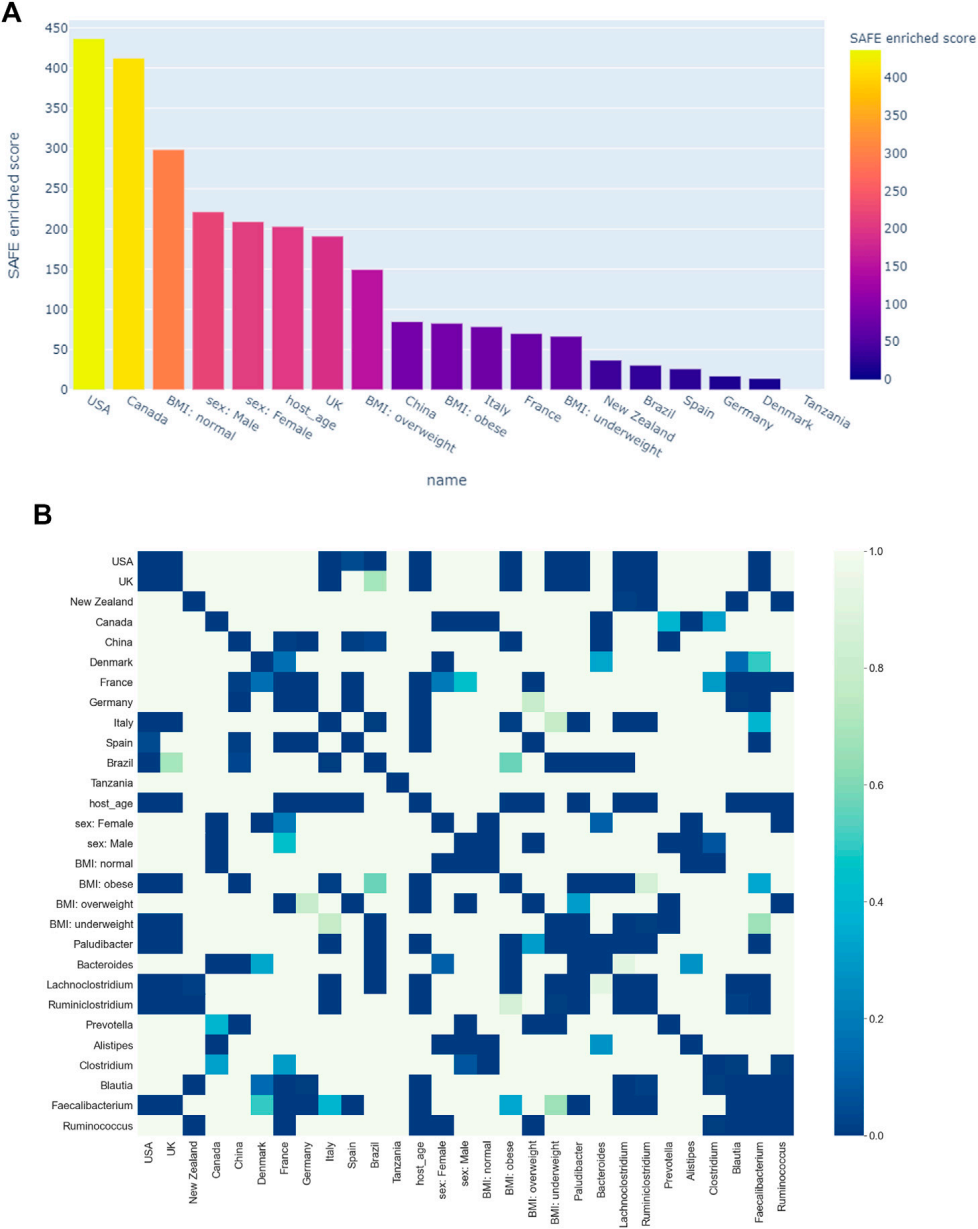
**(A)** Ranking of host metadata by their SAFE enriched score. **(B)** Co-enrichment significance values of host metadata and the most enriched taxa, presented as a heatmap. The significance threshold was set at the 0.5^th^ percentile threshold of all values.

We also explored the enrichment patterns of different taxa with host metadata. Figure 2B shows the taxa with the most enriched nodes in one figure, while Figures 5, 6 show network heat maps of the most relevant taxa. The top enriched taxa belong to the Bacteroidetes and Firmicutes phyla. Figure 6B shows a heatmap on the matrix of all co-enrichment pairs between host metadata and taxa of interest. Note that the significance threshold for co-enrichment was set to the 0.5th percentile of all scores, so that some of the seemingly lowest significances don’t pass the threshold.

Of the Bacteroidetes, *Bacteroides* is the genus with the second most enriched nodes, has a SAFE enriched score of 256, and is enriched in cluster one (Figure 2B). Looking at its heat map, it also becomes apparent that it is enriched in the junction of the two large clusters (Figure 5A). Despite the co-enrichment observable in these figures, no co-enrichment passes the significance threshold. Figures 2B,5B show that the genus *Prevotella*, which has one of the highest numbers of enriched nodes despite a relatively low SAFE enriched score of 99, is highly enriched exclusively in cluster 3, while it is abundant across the network. Although the heat map indicates co-enrichment of this genus with many variables such as China, Canada, or the US, they don’t pass the significance threshold. The genus with the most enriched nodes, *Paludibacater*, is the third most enriched taxon in the dataset, with a SAFE enriched score of 273. It is mainly enriched in the lower half of cluster 2 (Figures 2B,5C). Further, it is significantly co-enriched with the countries USA and Italy, as well as obese BMI, and *Lachnoclostridium*. *Alistipes* is exclusively enriched in the top left part of cluster 1 (Figure 6A). It is significantly co-enriched with Canada and normal BMI, which notably also co-enriched with each other.

The two genera with the most enriched nodes - *Lachnoclostridium* (SAFE enriched score 295) and *Ruminiclostridium* (284) - are both members of the *Clostridiales* order in the Firmicutes phylum and enriched predominantly in cluster 2 (Figures 6B,C), *Ruminiclostridium* particularly in the top half. Both are significantly co-enriched with the United States, United Kingdom, and Italy, *Ruminiclostridium* is further significantly co-enriched with host age. They are also both significantly co-enriched with *Faecalibacterium prausnitzii* and each other. Finally, the sparse and disconnected nodes in the top left of the network are most enriched by *Blautia* (SAFE enriched score 153) and *Faecalibacterium prausnitzii* (SAFE enriched score 223). *Blautia* is significantly co-enriched with New Zealand and *Ruminococcus*, while *F. prausnitzii* is significantly enriched with the UK and host age. Additionally, France, enriched across this sparse area, is significantly co-enriched with *Eubacterium, Ruminococcus,* as well as *Dorea* – a genus also significantly co-enriched with New Zealand.

## Discussion

Using TDA, we highlight novel differences and similarities of the gut microbiome across 12 countries. The TDA approach demonstrates the overlap between countries, a finding not possible with conventional approaches. We found distinct distributions of the countries across the TDA network that, through co-enrichment analysis, corresponded to the distribution of specific driver microbial genera, namely *Paludibacter*, *Bacteroides*, *Prevotella*, and *Alistipes* of the phylum Bacteroidetes, as well as *Lachnoclostridium* and *Ruminiclostridium*, as well as *Blautia, Faecalibacterium prausnitzii*, *Dorea*, *Eubacterium*, and *Ruminococcus* of the Firmicutes phylum. This highlights the potential utility of TDA as a complementary tool in microbiome research, and particularly of the library *tmap* as a helpful tool for implementing TDA in the microbiome space.

### Geographical Co-enrichment of Taxa

Broadly, TDA shows the similarities between the countries within each cluster. For example, the first cluster (Cluster 1) shows the shared features of the gut microbiome of Canada, China, Denmark, and Spain. Likewise, several countries have shared features in cluster 2 (USA, UK, Italy, New Zealand, Germany, Brazil), and Cluster 3 (USA, China, Canada). Importantly, the distinct clusters are associated with differences in the gut microbiome composition between these regions. It is also notable that membership of the cluster is not exclusive. For instance, the gut microbiome from Canadian samples shares features with cluster 1 and cluster 3. In contrast, the UK appears only in cluster 2. It is noteworthy that countries in close geographical proximity such as the USA and Canada, or France and Germany, seem to have important differences in their microbiome composition, whereas countries that are separated by thousands of miles, such as the UK and USA, share many features, as evidence by their co-enrichment. Below, we explore these differences in more detail with a focus on the specific co-enriched taxa and potential underlying explanations and confounds.

### Bacteroidetes

One of the most enriched genera this study identified is *Bacteroides* of the Bacteroidetes phylum. It was most enriched at the junction of clusters 1 and 2 and visually co-enriched with China, the USA, Denmark, and Brazil, although on individual testing these did not reach significance. The genus contains many pathogens and pathobionts and generally has a high virulence potential, as well as the highest antibiotic resistance of microbial genera (Wexler, 2007). It is further associated with diseases such as Irritable Bowel Disease (IBD; Walters et al., 2014) or the gut microbiome changes seen in ulcerative colitis carcinogenesis, as shown in a recent mouse study (Wang et al., 2019). Additionally, the genus *Bacteroides* is associated with obesity (Ppatil et al., 2012), which corresponds to the co-enrichment of an obese BMI score in cluster 1, which however was not statistically significant. Increased *Bacteroides* is associated with a long-term high-fat diet, specifically an omnivorous diet high in protein and animal fat (Wu et al., 2011; Zimmer et al., 2012; Ferrocino et al., 2015; Franco-De-Moraes et al., 2017). This diet is prevalent in high-income countries such as the USA and Canada (Ritchie and Roser, 2019), and becoming more common in middle-income countries, such as China and Brazil, as average income rises (Fu et al., 2012). Our results mirror the results of the tmap study by Liao et al. observed (2019), as they also associated the USA with a *Bacteroides* enterotype. While this overlapping result may be explained by the inclusion of data from the American Gut Project (McDonald et al., 2018) in this study, the data here shows more complex associations, owing to the increased number of included countries in our study. *Bacteroides* has also been specifically associated with an “industrialised” diet: a study comparing children from the USA and Egypt associated the American children with a *Bacteroides* enterotype, meaning a microbiome profile dominated by *Bacteroides* (Shankar et al., 2017). The Egyptian children on the other hand, who ate a Mediterranean diet rich in plant-based foods and fibres, were associated with the *Prevotella* enterotype.

Other studies find similar results for *Prevotella*: it has been associated with a long-term diet high in carbohydrates (Wu et al., 2011), and is particularly abundant in vegans (Franco-De-Moraes et al., 2017). In this study, *Prevotella* is enriched in cluster 3 as the main driver taxon. The cluster is disconnected from the other clusters and highly enriched with samples from China, Canada, and the USA, although the visually observed co-enrichment with *Prevotella* does not reach significance. The influence of diet, particularly vegan versus omnivorous, needs to be addressed in future studies of population-level microbiome studies and may have specific impacts on disease phenotypes.

*Paludibacter* is a fermentative genus that includes species producing the SCFA propionate (Qiu et al., 2017). While there is a lack of literature exploring this genus in humans, one study has associated it with a high fibre diet as it consumes mostly polysaccharides and was found to be abundant in children from rural Burkina Faso (De Filippo et al., 2010). Its statistically significant co-enrichment with the USA and Italy, as well as with obese BMI, is thus surprising. However, it should be noted that the abundance of *Paludibacter* is zero for the entirety of cluster 1, implying that its high abundance and enrichment in cluster 2 could be an artefact of the genera sampled in the different studies.

*Alistipes* is another genus of the Bacteroidetes phylum, of the *Parabacteroides* family, that was among the most highly enriched taxa. Specifically, it was enriched in the top half of cluster 1, and significantly co-enriched with Canada and normal BMI, which also co-enrich with each other. This association is in line with previous research finding an association between *Alistipes* and a lower BMI (Aguirre et al., 2016; Lv et al., 2019). The association with Canada on the other hand could be a sampling artefact, as Canada has a particularly high number of normal BMI samples compared to other countries in this study. Further, 77% of the Canadian sample used here are of normal BMI, while the Canadian adult population has an obesity rate of 64% (Government of Canada, 2017). Additionally, studies have found *Alistipes* to have both beneficial, as well as detrimental effects on the host: on the one hand, it has been found to attenuate colitis in mice (Dziarski et al., 2016), but on the other hand, it has consistently increased abundance in Parkinson’s Disease (PD) patients (Barichella et al., 2016; Bedarf et al., 2017; Li C. et al., 2019). This could be further explored by applying our approach to the gut microbiome of populations with a differing prevalence of PD.

### Firmicutes

Two of the top enriched taxa were Firmicutes of the order Clostridiales: *Lachnoclostridium* and *Ruminiclostridium.* While the phylum Firmicutes, and specifically the order Clostridiales, is often associated with beneficial effects for the host, as it contains many SCFA producers (Morrison and Preston, 2016; Levy et al., 2017), these two genera seem to fall out of this pattern.

Similar to the above *Bacteroides*, *Lachnoclostridium* has been associated with the changes in gut microbiome found in the carcinogenesis of ulcerative colitis in mice (Wang et al., 2019) - but recovered to a normal abundance after probiotic treatment. Additionally, a new *Lachnoclostridium* species has recently been found to contain a specific genetic marker that is enriched in people with colorectal adenoma, leading to it being suggested as a non-invasive diagnostic marker of the disease (Liang et al., 2020).

*Ruminiclostridium* is a medium-chain fatty acid (MCFA) producer. MCFAs, such as Caproic acid (CA), are metabolites that are less studied than SCFAs. There is evidence that MCFAs antagonise the anti-inflammatory effects of SCFAs by enhancing TH1 and TH17 cell differentiation in a CNS autoimmune model (Haghikia et al., 2015). Additionally, CA was found to be augmented in Multiple Sclerosis patients while SCFAs were reduced, correlating with an immunological profile of an increase in TH1 and TH17 and a decrease in Treg lymphocytes (Saresella et al., 2020). *Ruminiclostridia* were also elevated in a mouse model of dysbiosis – and intriguingly also increased in aged mice (Liu et al., 2020). This is relevant as in this study, *Ruminiclostridium* was significantly co-enriched with host age.

It thus seems that these two Firmicutes are both associated with pro-inflammatory properties. These genera were highly enriched in cluster 2 and specifically co-enriched significantly with the USA and UK, suggesting an important role in those countries’ microbiome profile that is distinct to that of other countries. This is further supported by the significant co-enrichment between the USA and UK, as well as between *Lachnoclostridium* and *Ruminiclostridium.* This finding is opposite to that of Liao and colleagues (2019) who found the two countries to have distinct microbiome signatures, specifically that only the UK but not the USA were co-enriched with the family Ruminococcaceae, which contains *Ruminiclostridium*.

Finally, the sparse and disconnected collection of nodes in the top left of the TDA network is highly enriched with France and five different genera of the order Clostridiales: *Blautia*, *Faecalibacterium prausnitzii*, *Dorea*, *Eubacterium*, and *Ruminococcus*, the last three of which were statistically significantly co-enriched with France. Some were also associated with the geographically separated New Zealand. The Clostridiales order has been identified as one of the main SCFA producers in the human gut and indeed all of these genera have been previously identified as butyrate producers (Venegas et al., 2019). However, the fact that the nodes of this network area do not cluster together complicates adequate interpretation of this finding, warranting further investigation by future studies.

### Limitations

This study has several limitations. Firstly, the microbiome data is assumed to be representative of the country population, which may not be the case if sampling bias is present. The importance of this is highlighted in the unexpected co-enrichment between normal BMI and Canada, which is likely an artefact of sampling. Similarly, other countries such as Denmark, Spain, or Tanzania, are missing many data points on BMI, limiting the conclusions that can be drawn from BMI enrichment. Secondly, diet could not specifically be controlled for, given the dataset is a composite of many studies and diet was not collected for all samples. Similarly, the clear separation of enrichment between cluster 1 and 2 for the most highly enriched taxa might be an artefact of the way the data was curated as not all countries sequenced exactly the same taxa. However, this is unlikely to be a significant confounder, apart from the *Paludibacter* enrichment, as the heatmaps show taxa can be highly abundant but not highly enriched across the network. Another potential limitation is using microbiome data at the genus level. Each genus is composed of many species which in turn can be made up of various strains, all having potentially different effects. While not all strains constituting a genus are fully sequenced yet, analysis at species-level could still aid in making interpretation more precise. As species-level data is also available from the GMrepo database, this could be a future extension of the current study.

## Conclusion

In summary, we find that TDA highlights novel insights into the differences and similarities between the gut microbiome at a population level, both between geographically separated countries and within single countries. This underscores the importance of accounting for factors such as geography or regionally varying factors such as diet when conducting microbiome studies. Further, the dimensionality preserving TDA approach may yield more depth and a richer understanding of the changes in the gut microbiome seen across several diseases and clinical phenotypes that would not be possible using conventional approaches. The python library *tmap* seems to serve as a valuable vehicle for such analyses, particularly due to the inclusion of co-enrichment analysis and network visualisation. TDA may be particularly beneficial for patient data, as current studies cannot account for non-linearity which is often present in such data. This would, however, require larger datasets on clinical phenotypes than are currently available in curated datasets such as GMrepo (Wu et al., 2020) or MGnify (Mitchell et al., 2020). Finally, there is the potential for TDA to be integrated with machine learning approaches, a novel avenue of research (Hensel et al., 2021). This may identify specific taxa for interventional therapeutics, though there are significant barriers to overcome before this may be feasible.

## Data Availability

A publicly available dataset was analyzed in this study, which can be found here: https://gmrepo.humangut.info/home. The code used to obtain the results of this article can be found here: https://github.com/thesharmalab-team/tmap_geography.
